# Supplementary material for: Rapid Identification of Druggable Targets and the Power of the PHENotype SIMulator for Effective Drug Repurposing in COVID-19
Source: Res Sq. 2021 Apr 14:rs.3.rs-287183. Preprint. [Version 1] doi: 10.21203/rs.3.rs-287183/v1 (PMC8057245; doi:10.21203/rs.3.rs-287183/v1)
Supplement: Supplement [file 5c304e807a880ec94a569d14.docx]

**SUPPLEMENTAL INFORMATION**

**Rapid Identification of Druggable Targets and the Power of the PHENotype SIMulator for Effective in Silico Drug Repurposing in COVID-19**

Naomi I. Maria, Rosaria Valentina Rapicavoli, Salvatore Alaimo, Evelyne Bischof, Alessia Stasuzzo, Jantine A.C. Broek, Alfredo Pulvirenti, Bud Mishra, Ashley Duits*,* Alfredo Ferro

**Supplemental Figures:**

Figure S1, related to Figure 2

Figure S2, related to Figure 2

Figure S3, related to Figure 2

Figure S4, related to Figure 3

Figure S5, related to Figure 4

Figure S6, related to Figure 4

**Supplemental Tables:**

Table S1, related to Methods

Table S2, related to Figure 3 and Figure S4

**Additional Supplemental Material**

Pathway analyses for *1)* MITHrIL, *2)* SPIA, *3)* Reactome Pathways and *4)* Gene Ontology Enrichment analysis for all host cells assessed.

**Supplemental Figures**

**Figure S1**, ***related to Figure 2C.*** PHENSIM *in silico* predicted average

pathway perturbation is depicted in heatmap for top signaling pathways

activated by SARS-CoV-2, as identified by Catanzaro *et al*. Results are depicted in normal human bronchial epithelial (NHBE) cells, cultured human airway epithelial cells (Calu-3) cells, and A549 lung alveolar cells­ transduced with human ACE2 (A549-ACE2), which enabled apparent SARS-CoV-2 replication *in vitro* at low multiplicity of infection (MOI) 0.2. Select pathways are highlighted in detail.


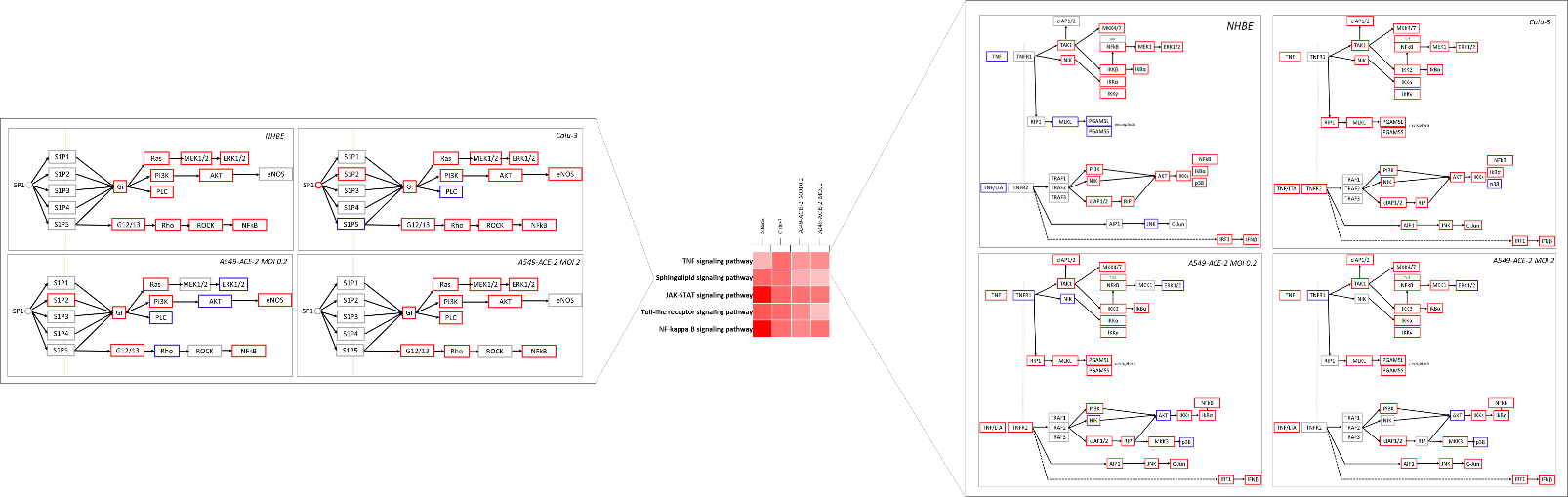

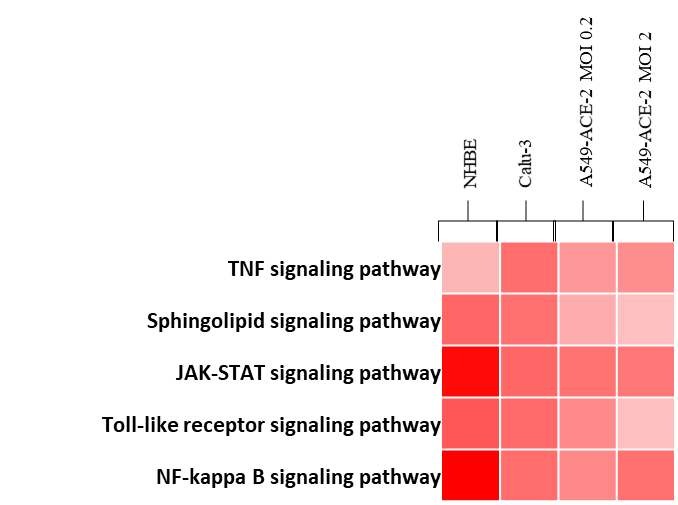


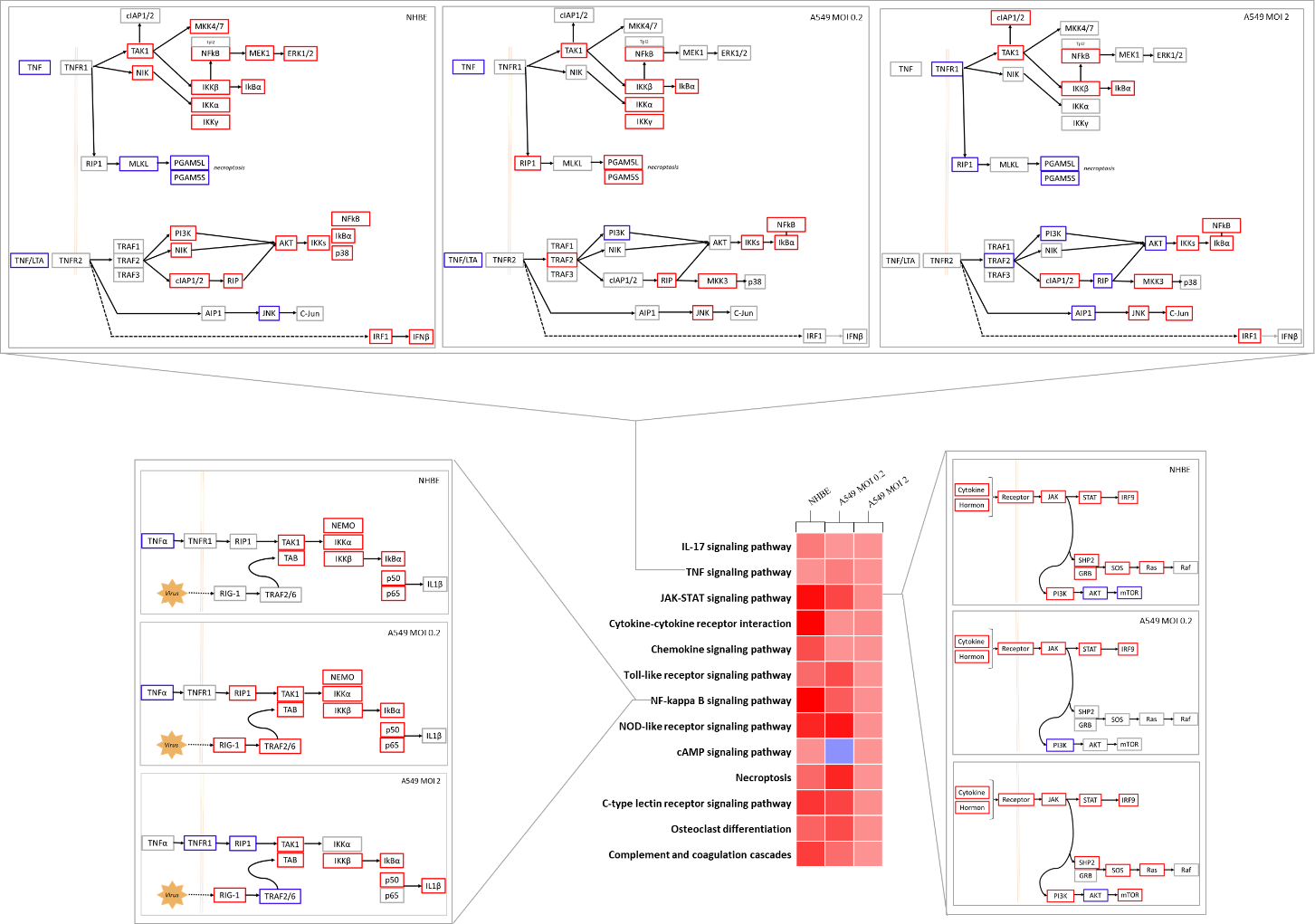


**Figure S2, *related to Figure 2C*.** PHENSIM *in silico* predicted average pathway perturbation is depicted in heatmap for top signaling pathways activated by SARS-CoV-2, as identified by Draghici *et al.,* using the GEO dataset GSE147507. Results are depicted in normal human bronchial epithelial (NHBE) cells, and A549 lung alveolar cells­ (without human ACE2) at low, which enabled apparent SARS-CoV-2 replication *in vitro* at low multiplicity of infection (MOI) 0.2. Select pathways are highlighted in more detail on gene level.


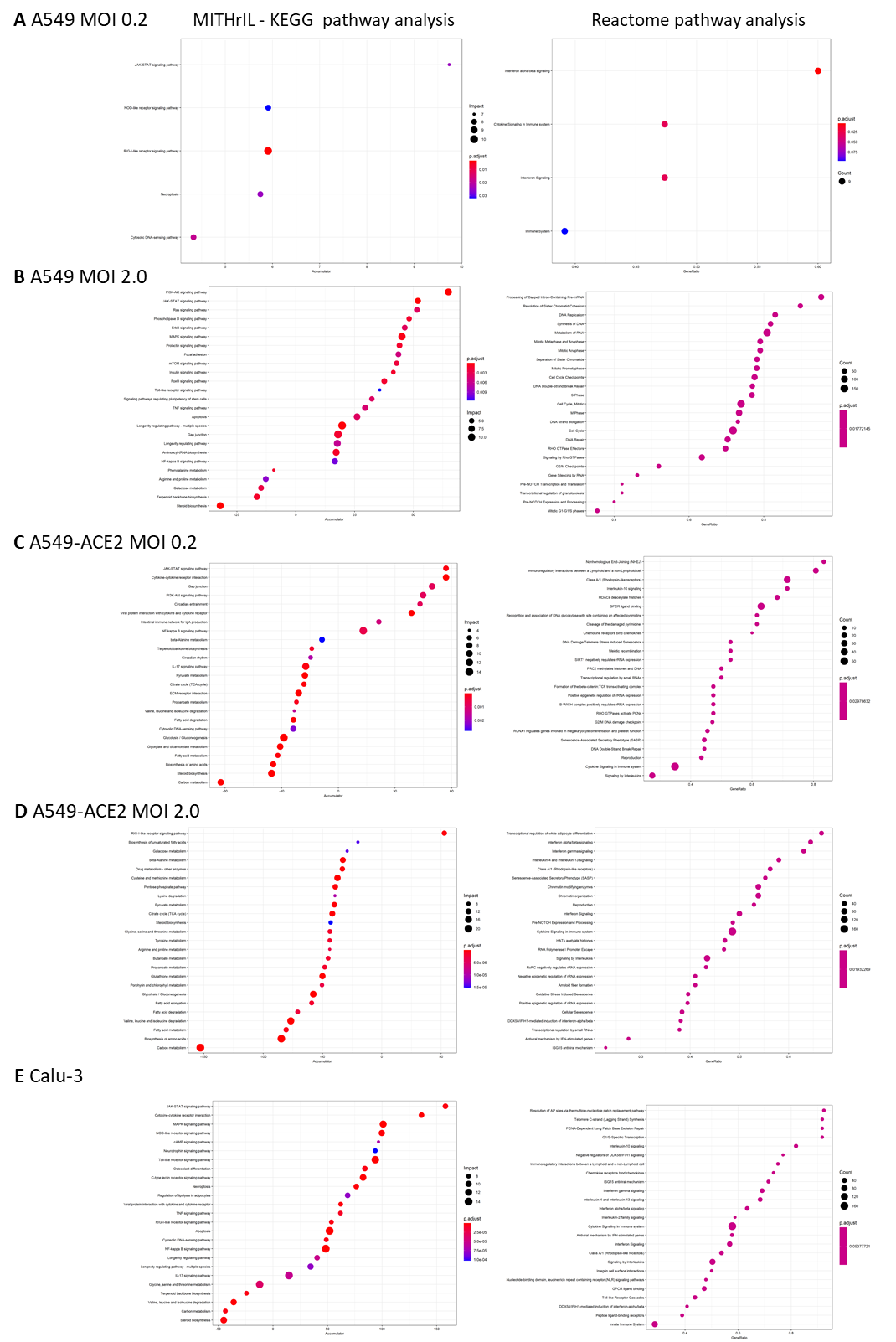


**Figure S3**, ***related to Figure 2D&E.***

MITHrIL (using KEGG pathways; left) and Reactome pathway analysis (right) was used to assess top meta-pathways for A549 lung alveolar cells­ +/- transduction with human ACE2 (A549-ACE2), at low and high multiplicity of infection (MOI), and in cultured human airway epithelial (Calu-3) cells. according to impact (circle size) and significance (color-gradient for adjusted p-value). The accumulator is the accumulation of all perturbations computed for that particular pathway.

**
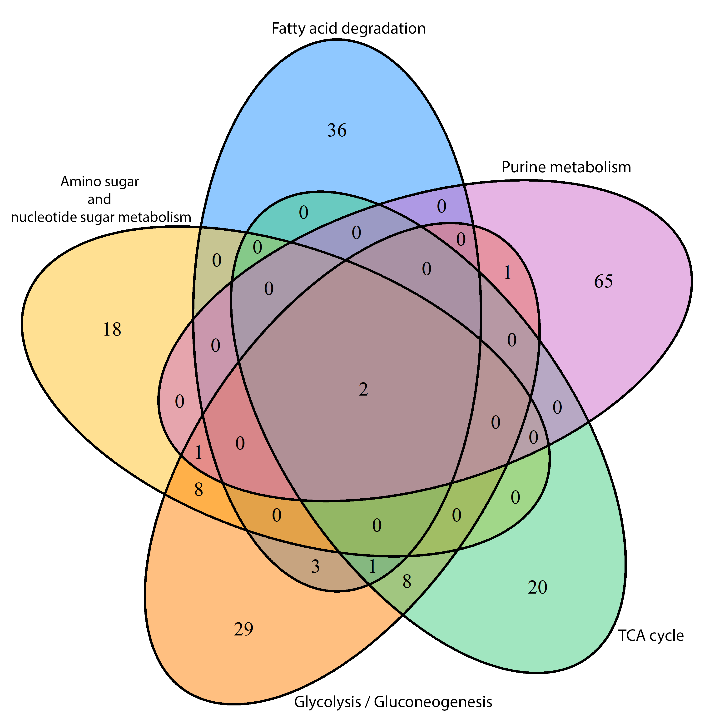

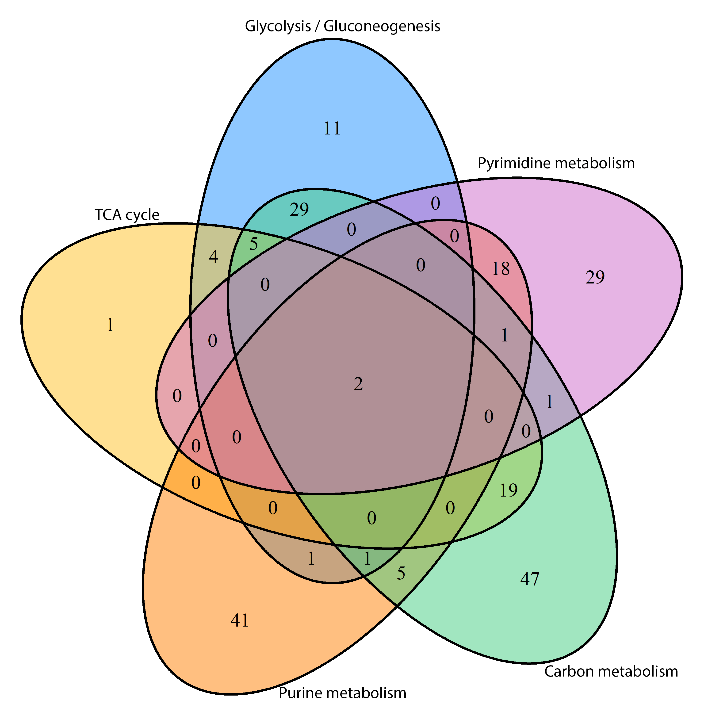
Figure S4. Venn diagrams of the perturbed genes of the significant metabolic pathways, related to Figure 3 and Table S2.** This figure illustrates the Venn diagrams obtained by calculating the intersections between the perturbed genes of the metabolic pathways identified in the PHENSIM simulation for the proteomics data illustrated in Figure 3. The analysis was conducted to exclude the hypothesis that a common core of altered enzymes was driving the significant perturbation of these closely related metabolic pathways. To construct the graphs, we took all the metabolic pathways considered essential for the infection in Bojkova et al. 2020; FDR-adjusted p-value < 0.05 (Table S2). For each of these pathways, the perturbed genes were determined according to the PHENSIM activity score. Finally, all possible intersections were calculated. To better visualize the results, the 7 pathways obtained with the previous criteria (see Fig. 3D) were divided into two groups of 5 pathways: (***left***) Fatty acid degradation, Amino sugar and nucleotide sugar metabolism, Glycolysis /Gluconeogenesis, Citrate cycle (TCA cycle), and Purine metabolism; (***right***) Glycolysis /Gluconeogenesis, Citrate cycle (TCA cycle), Purine metabolism, Carbon metabolism, and Pyrimidine metabolism.

**
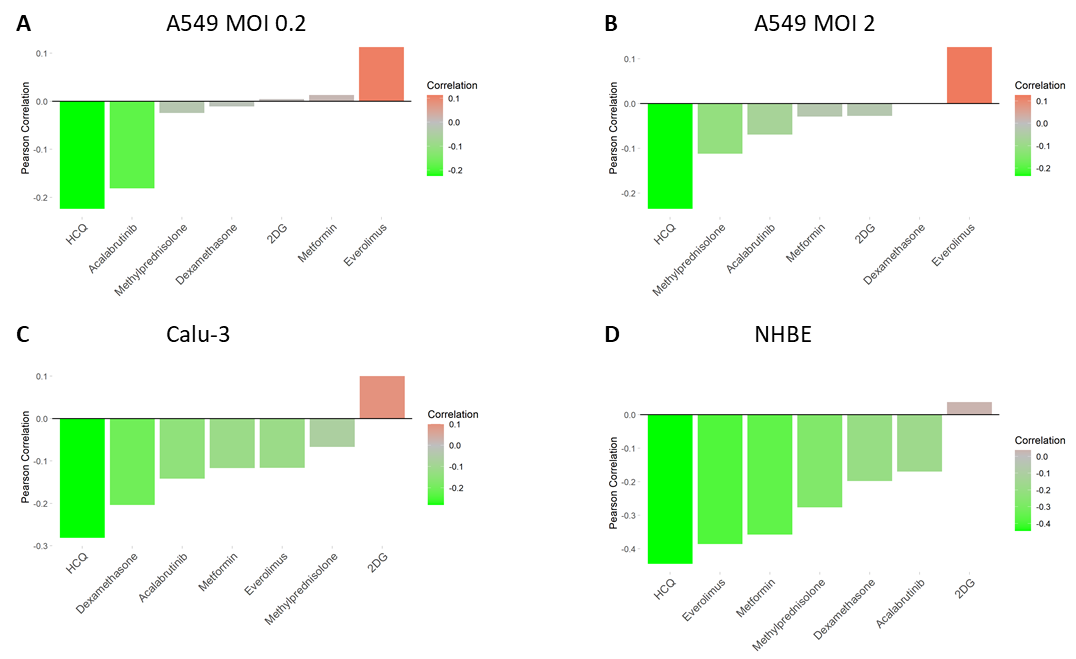
Figure S5. Drug repositioning candidates for COVID-19 as predicted by PHENSIM, *related to Figure 4*.** The PHENSIM drug strategy approach to test candidate drugs for potential repurposing for COVID-19 treatment is depicted for the remaining cell type scenarios tested**; A)** A459 at low MOI 0.2 and **B)** A459 at high MOI 2 (without ACE2 expression). **C)** Calu-3 cells and **D)** NHBE cells. Drug candidates represented here: Methylprednisolone, Metformin (mTOR-inhibitor), (Hydroxy)chloroquine (HCQ-CQ), Acalabrutinib (BTK-inhibitor), 2-Deoxy-Glucose (2DG) and Everolimus (mTOR-inhibitor). ACE2, angiotensin-converting enzyme; MOI, multiplicity of infection.


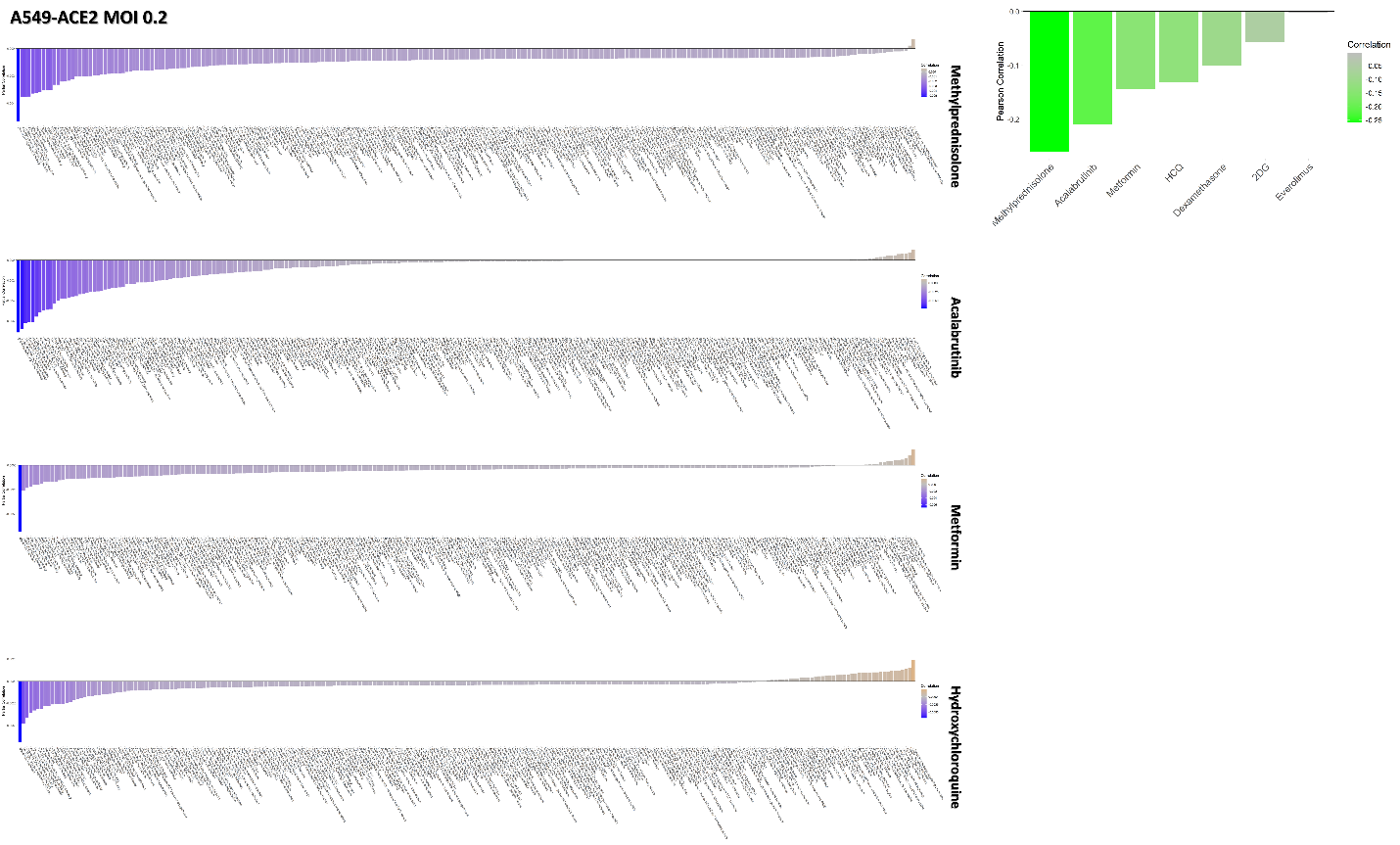

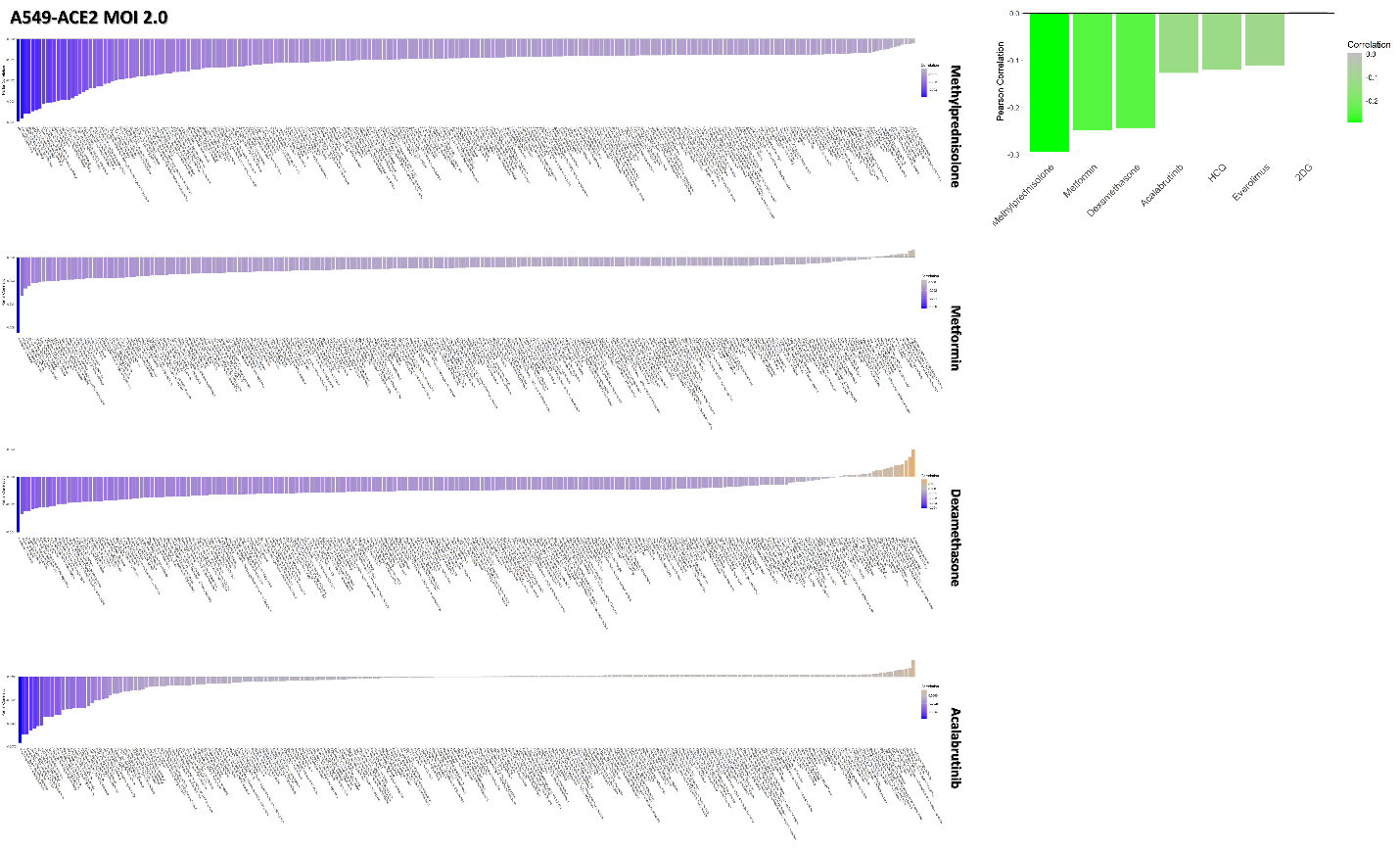
**Figure S6. Top drug repositioning candidates as predicted by PHENSIM, *related to Figure 4*.** Top 4 candidates for A549-ACE2 MOI 0.2 (top) and A549-ACE2 MOI 2.0 (bottom). Methylprednisolone, Acalabrutinib (BTK-inhibitor), Metformin (mTOR-inhibitor), (Hydroxy)chloroquine (HCQ-CQ), Dexamethasone. ACE2, angiotensin-converting enzyme; MOI, multiplicity of infection.
